# A Case Report of Hematemesis in a 4‐Week‐Old Neonate: Clinical Reasoning and Outcome After Empiric Acid Suppression

**DOI:** 10.1155/crpe/6283741

**Published:** 2026-08-03

**Authors:** Nora Lyang, Daisy Ward, Amy Phu, Yadira Arguelles, Thiagarajan Nandhagopal

**Affiliations:** ^1^ College of Osteopathic Medicine of the Pacific, Western University of Health Sciences, Pomona, California, USA, westernu.edu; ^2^ Department of Pediatrics, Kern Medical Center, Bakersfield, California, USA, kernmedicalcenter.com

**Keywords:** neonatal hematemesis, proton pump inhibitor, upper gastrointestinal bleeding

## Abstract

**Introduction:**

Neonatal hematemesis is an uncommon presentation with a broad differential diagnosis ranging from benign to life‐threatening etiologies, and there are no well‐established, evidence‐based management guidelines. This case highlights the diagnostic challenges and potential role of empiric acid suppression in a clinically stable neonate.

**Case(s) Presentation:**

A 4‐week‐old term male with a prior episode of coffee‐ground emesis at 3 days of life presented with recurrent hematemesis consisting of bright red blood. He was hemodynamically stable with an unremarkable physical exam. Laboratory evaluation demonstrated stable hemoglobin, mildly elevated bilirubin, and no evidence of coagulopathy. Imaging, including abdominal ultrasound and radiographs, was unremarkable. Infectious and hematologic workups were negative. Esophagogastroduodenoscopy revealed superficial gastric mucosal abrasions without active bleeding, ulcers, or structural abnormalities. The patient was managed initially with intravenous acid suppression and supportive care, and then transitioned to oral lansoprazole. He tolerated feeds without recurrence of hematemesis and was discharged with a 14‐day course of proton pump inhibitor therapy. At two‐week follow‐up, he remained asymptomatic with normal feeding and no further bleeding episodes.

**Conclusion:**

This case underscores the importance of systematic evaluation in neonatal hematemesis and suggests that empiric proton pump inhibitor therapy may be a reasonable option in select stable patients with suspected mucosal injury.

## 1. Introduction

Hematemesis in the neonatal population may be due to a wide spectrum of differential diagnoses that can range from benign to acutely emergent etiologies. Upper gastrointestinal bleeding (UGIB) is defined as bleeding proximal to the ligament of Treitz and is uncommon in neonates but may present with coffee ground material vomit, gross blood emesis, tarry stools, or even hematochezia [1]. Gastric lesions causing hematemesis are relatively common among neonates admitted to intensive care units, occurring in approximately 20% of all admissions. Notably, a significant proportion of these affected infants remain asymptomatic, without any clinical evidence of UGIB [2]. There currently lacks a widely accepted treatment guideline for neonatal UGIB, with treatments currently targeted toward stabilizing the patient and symptomatic treatment. We present a case of a term neonate with a history of hematemesis at three days old who developed a second episode of hematemesis at four weeks old without a known inciting perinatal event, treated successfully with a 14‐day course of lansoprazole.

## 2. Case Presentation

A 4‐week‐old Hispanic male (Ht: 53 cm, Wt: 4.51 kg) born at 37 weeks gestation by normal spontaneous vaginal delivery to a 34‐year‐old G8P7017 mother presented to the ED for hematemesis and jaundice. The patient had a history of hematemesis at 3 days of age where orogastric tube revealed a large amount of coffee‐ground aspirate. Subsequent lavage revealed clear aspirates and normal intestinal gas patterns on abdominal X‐ray. The patient stayed in the NICU for hematemesis management for 8 days where he was treated symptomatically after having negative sepsis, hematologic, and metabolic workup. He was also treated with phototherapy for incidental hyperbilirubinemia and discharged on feeding with maternal breast milk.

Three weeks after NICU discharge, the patient became increasingly fussy for one day and had three episodes of hematemesis with blood. The patient’s mother reported that the first episode consisted of approximately one tablespoon of bright red blood, followed by a second episode with a smaller volume, and a third episode of blood‐tinged emesis mixed with milk and small clots. The mother reported that the patient had been having nonbilious, nonprojectile, milky emesis around three times a day and continued to look jaundiced since being discharged from the NICU. She denied any family history of bleeding disorders, coagulation disorders, and/or gastrointestinal (GI) disorders. The mother has Type A+ blood and reported good prenatal care; negative screening labs; prenatal vitamin intake; and no tobacco, alcohol, or illicit drug use. The patient had an uncomplicated delivery and received vitamin K at birth and had been exclusively breastfeeding every 2 h with normal stooling and voiding patterns.

Upon arrival at the emergency department, the patient’s vitals were stable and physical exam was unremarkable. IV fluids, 1.1‐mg famotidine IV, and 0.5‐mg vitamin K were given to the patient. Labs revealed alkaline phosphatase 545 IU/L, AST 49 IU/L, total bilirubin 12.8 mg/dL (0.7 mg/dL direct), and PT 15.3 s and PTT 36.2 s. Hemoglobin was stable at 11.8 g/dL, and CBC revealed occasional helmet cells.

An abdominal ultrasound revealed trace right pelvocaliectasis, but otherwise no acute findings. Blood culture exhibited no growth, and urine culture grew *E. coli*. Fibrinogen, D‐dimer, Factor VII, Des‐γ‐carboxy‐prothrombin (DCP/PIVKA), and GGT were all within normal limits. During the hospital stay, the patient vomited clear liquid once and had one episode of melena while vitals remained stable.

The patient was started on omeprazole and subsequently transferred to Valley Children’s Hospital (VCH). At VCH, the patient was started on IV esomeprazole 0.5 mg/kg dose (2.16 mg) BID, continued as NPO, and placed on IV D5 NS maintenance fluids. The next morning, an esophagogastroduodenoscopy (EGD) was performed to visualize the upper GI tract and was notable for superficial abrasions in the body of the stomach, without any ulcers or active bleeding noted. Ultrasound and X‐ray of the abdomen were normal, without any free air or obstruction. CBC/CMP drawn were normal, except the patient continued to have an elevated indirect bilirubin (11.1 mg/dL). MRSA culture was negative. Breastfeeding was resumed, and the patient tolerated feeds without further emesis or blood in spit‐up. Vitals remained stable with a reassuring physical exam, and the patient continued to produce appropriate stool and urine output.

The next day, the patient remained stable with a reassuring physical exam and vitals and was deemed ready for discharge. The patient was prescribed a 14‐day course of lansoprazole 9 mg once a day and had a scheduled follow‐up with his GI and pediatrician. At a two‐week phone call follow‐up, the patient had no recurrence of hematemesis and was continuing to feed well on breast milk with normal stooling patterns and no alcoholic stools. No adverse effects from the 14‐day PPI therapy were reported or observed.

## 3. Discussion

Hematemesis in neonates is a relatively uncommon presentation that encompasses a range of etiologies that must be carefully considered to identify the underlying cause. In light of the patient’s prior episodes of hematemesis, particular attention was directed toward assessing the temporal association between feeding and symptom onset that led to recurrent hematemesis in the absence of clinical instability.

Hematologic causes such as coagulopathies were investigated as a possible cause of this patient’s hematemesis but were found to be less likely, given the patient’s stable INR, absence of family history of bleeding disorders, and reasonable Factor VII levels. Consumptive coagulopathies were similarly found to be improbable since the patient had normal platelet levels and a reassuring D‐dimer and fibrinogen level. Late‐onset vitamin K deficiency bleeding was considered, particularly in the context of exclusive breastfeeding; however, the administration of prophylactic vitamin K at birth, stable INR, and only mildly elevated PT/PTT values made this diagnosis less likely. The patient’s indirect hyperbilirubinemia observed was most consistent with breast milk jaundice, which showed gradual improvement during hospitalization.

Hepatic etiologies, including portal hypertension, were evaluated but deemed unlikely given the patient’s young age, normal liver enzyme profile, stable INR, normal GGT and DCP/PIVKA, and unremarkable abdominal ultrasound findings. Common causes of neonatal hematemesis, such as swallowing of maternal blood, were also less likely, as the mother denied any mastitis, irritation, or bloody discharge. GI causes such as necrotizing enterocolitis and malrotation were considered but deemed unlikely given the normal abdominal radiograph, absence of abdominal distension, and lack of tenderness on palpation. Meckel’s diverticulum and pyloric stenosis, though possible, were considered less likely in a neonate of this age and lack of projectile vomiting.

The differential diagnosis for hematochezia in this infant remains broad but may be most likely due to potential GI causes such as orogastric tube lavage–induced trauma, prolapse gastropathy syndrome, or Mallory–Weiss tears secondary to recurrent emesis, gastritis, and vascular malformations such as Dieulafoy lesions [1]. Upper endoscopy showed only superficial abrasions in the body of the stomach, ruling out other GI causes such as gastric or duodenal ulcers, esophageal damage, and pyloric stenosis, even though neonatal GI bleeding has often been found to be associated with mucosal ulcers [3]. Thus, we hypothesize that the patient’s feeding intolerance and recurrent emesis may have been driven by an underlying food protein–mediated process, such as food protein–induced enterocolitis syndrome (FPIES) or cow’s milk protein allergy (CMPA), with repetitive vomiting leading to the superficial gastric mucosal injury observed on endoscopy and ultimately resulting in hematochezia.

The decision to discharge the patient on lansoprazole was most likely reserved for this clinical scenario [4]. Systematic reviews and guidelines show that PPIs and H2 receptor antagonists can increase gastric pH and may reduce the duration or recurrence of upper GI bleeding in infants, but these benefits have not consistently been reflected in improved symptoms or major clinical endpoints such as mortality or need for transfusion [4–6]. PPIs are commonly prescribed for pathological GERD in neonates [7], but there is a lack of robust neonatal data on the use of PPIs for upper GI bleeding, which points to the dire need for further studies to clarify the guidelines surrounding treatment [8–11]. In this case, the use of lansoprazole in this patient was carefully weighed in a risk‐benefit assessment and prescribed in an attempt to protect the gastric mucosa and reduce acid‐related injury and bleeding. To this date, the patient has finished his course of lansoprazole and has not had any further hematemesis episodes, validating the choice to prescribe a PPI for his case.

**FIGURE 1:**
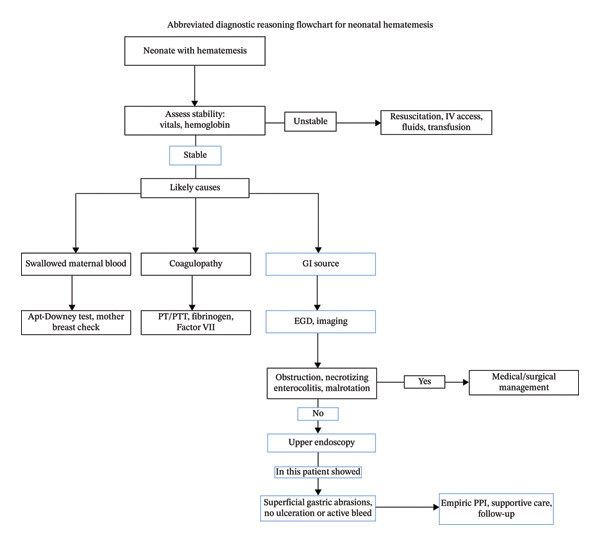
Abbreviated diagnostic reasoning flowchart for neonatal hematemesis proposed clinical reasoning framework for the evaluation of neonatal hematemesis, outlining potential etiologies and diagnostic considerations. The pathway illustrates how clinical findings and diagnostic testing guided management and supported the treatment with proton pump inhibitor therapy.

Upper GI bleeding is an uncommon presentation of neonates. In a case–control study of 5180 healthy full‐term infants, 64 (1.23%) developed UGIB within the first 4 days of life [3]. Among the 53 infants who underwent endoscopy, gastric or duodenal ulcers represented approximately 34% of the cases. The remaining cases showed gastric erosions (nonulcerative mucosal breaks), esophageal lesions, or combinations thereof, similar to the patient presented in this case report [3, 12]. Pediatric‐specific data and treatment guidelines for these etiologies are limited, and there are currently no clear guidelines for neonatal UGIB [4, 5, 13], highlighting the need for further research. To aid clinicians in the initial evaluation, we propose an abbreviated diagnostic reasoning flowchart for neonatal hematemesis (Figure 1).

This case report has notable strengths as well as limitations. One strength of this report is the detailed diagnostic evaluation and documented resolution of hematemesis after treatment with proton pump inhibitor therapy. Another strength of this report is the critical evaluation performed to exclude other serious hematologic, hepatic, and structural GI causes of neonatal hematemesis. However, this report is limited by its nature as a single case, which restricts generalizability. In addition, a definitive etiology for the hematemesis could not be established despite this extensive evaluation.

## 4. Conclusions

Studies show that after initial stabilization and control of upper GI bleeding, a transition to oral PPI for up to 2 weeks may be considered, but this is based on limited evidence and primarily adult data [13]. This case illustrates the successful management of neonatal UGIB with a 14‐day course of lansoprazole, highlighting the potential applicability of the proposed adult treatment in the neonatal population.

## Funding

This research did not receive any specific funding.

## Ethics Statement

This manuscript was prepared following the CARE guidelines (https://www.care-statement.org).

## Consent

Written informed consent from the patient’s mother was obtained for publication of this case report and any accompanying images. A copy of the written consent is available for review by the Editor‐in‐Chief of this journal.

## Conflicts of Interest

The authors declare no conflicts of interest.

## Data Availability

The data that support the findings of this study are available from the corresponding author upon reasonable request.
